# Attractive Models: How to Make the Silicon Cell Relevant and Dynamic

**DOI:** 10.1002/cfg.205

**Published:** 2003-02

**Authors:** Hans V. Westerhoff, Frank Bruggeman, Jan-Hendrik S. Hofmeyr, Jacky L. Snoep

**Affiliations:** 1 Stellenbosch Institute for Advanced Study Stellenbosch 7600 South Africa; 2 Department of Molecular Cell Physiology BioCentrum Amsterdam Faculty of biology Vrije Universiteit, De Boelelaan 1087 Amsterdam NL-1081 HV The Netherlands; 3 Department of Biochemistry University of Stellenbosch Matieland 7602 South Africa


**Comparative and Functional Genomics**
*Comp Funct Genom* 2003; **4**: 155–158.
Published online in Wiley InterScience (www.interscience.wiley.com). **DOI:** 10.1002/cfg.205

**Conference Review**

### Attractive models: how to make the silicon cell relevant and dynamic

Hans V. Westerhoff[1,2]*, Frank Bruggeman[2], Jan-Hendrik S. Hofmeyr[3] and Jacky L. Snoep[2,3]

[1] *Stellenbosch Institute for Advanced Study, Stellenbosch 7600, South Africa*
[2] *BioCentrum Amsterdam, De Boelelaan 1087, NL-1081 HV Amsterdam, The Netherlands*
[3] *Department of Biochemistry, University of Stellenbosch, Matieland 7602, South Africa*

**Correspondence to:*
*Hans V. Westerhoff, Department of Molecular Cell Physiology, BioCentrum Amsterdam, Faculty of biology, Vrije Universiteit, De Boelelaan 1087, 1081 HV Amsterdam, The Netherlands.*
*E-mail: hw@bio.vu.nl*

Received: 10 July 2002
Accepted: 2 August 2002

### Is biology just a complicated special case of physics? Are its networks special?

The completion of the DNA inventory of a number of living organisms has invalidated the excuse that biology cannot be exact because it is necessarily incomplete. No longer can the failure of a model to explain experimental data be attributed to the interference of as yet unknown factors. Any such factor can become known given sufficient experimental effort. In addition, the concentrations of the transcripts of *all* the genes, of all proteins and soon of a number of organisms can now be measured. This brings cell biology irreversibly into the realm of the physical chemical sciences, where models and hypotheses are scrutinized for consistency with all known principles and experimental data, and then tested by specially designed critical experiments.

Does this mean that biology has become a branch of physics (and chemistry)? As functional genomics unfolds, it becomes clearer and clearer that it has not and will not. At least not when ‘physics’ and ‘chemistry’ are taken to refer to the archetypical sciences that were so successful in the last century. Physics and chemistry were successful in the selection or dissection of their objects of study. They only considered small, more or less autonomous parts, which were then studied independently. For quite a while (molecular) biology followed suit, also dissecting and then characterizing and understanding the individual macromolecules of living cells in isolation. And indeed, *that* (molecular) biology came close to physics and chemistry.

Biology is unique among the natural sciences in that it is ultimately bound to a single object of study, which is life itself, or at least to a single class of objects, i.e. living systems. And ‘life’ cannot be reduced indefinitely. There is a smallest unit of life: the autonomously replicating unit. This unit cannot be dissected further without losing that characteristic of biology, i.e. life. That is not to say that biology should not also dissect living systems and engage in molecular biology, but it should ultimately return to its object of study, i.e. living systems and life itself.

How large and complex is this minimum unit of biology? We shall address this question in three ways, from the insight of what may be needed for life, from genome sequence data and from gene

Copyright © 2003 John Wiley & Sons, Ltd.

expression data. The smallest autonomously replicating unit is the living cell. Biochemistry has already explained why this unit is indivisible. Biological information being stored in a sequence of reliably pairing bases requires the biosynthesis of those bases, the sugar phosphate backbone, as well as a supply of free energy (ATP). In order to drive the re-phosphorylation of ADP, catabolic pathways such as glycolysis are needed. For the rates of these processes to be competitive, protein catalysts (often with co-factors and prosthetic groups) are required. This necessitates a protein synthesis machinery. To keep everything together, a lipid bilayer is an appropriate device, provided that extra transport proteins are inserted [14].

The sequencing of entire genomes has confirmed this view that there is a substantial minimum size of the unit of life, where size is here measured in terms of the number of processes. The genome of *Mycoplasma genitalium* was shown to contain about 400 genes, of which only 100 or so may be dispensable. Following the 'first law' of biochemistry, that every process in living cells is catalysed by something that involves a protein, the 'first law' of genetics, that each process corresponds to a gene, and the 'first law' of molecular biology, that each gene corresponds to a stretch of nucleic acid, this suggests that a few hundred processes constitute the minimum unit of life. The experimental observation that deletion of any of the 300 processes interferes with the living state [4] constitutes the experimental proof that 300 processes is indeed the minimum required for life.

The second phase of genomics brought an additional suggestion of a minimum unit of life. External perturbations were seen to cause the mRNA expression levels of many genes to change [e.g. 2, 12]. In most studies of the transcriptome, a functional change appeared not to be accompanied by the altered expression of a single, 'key', gene, but rather by that of a multitude of genes.

A few hundred different processes involve hundreds of independent variables. If indeed this unit of hundreds of processes were not reducible, then this should make life unsuitable for the traditional paradigms of physics and chemistry. Life should be yet another one of those cases where (traditional) physics 'either stands mute, or gives the wrong answers' [8]. Yet the opinion of many physicists, chemists and molecular biologists has been that this schism between physics and biology was not essential: life was conceived as a collection of processes that were so involved in each other that no general principles ruled. Life should just be a complicated special case of physical chemistry. It should therefore be good enough to examine each of the processes individually. The grand total of the understanding of the 300 processes should then constitute the understanding of 'life'.

This point of view admits that biology as a science reaches beyond physics and chemistry, but then holds that the extension is trivial. Is the extension indeed trivial? Any non-trivial difference between the unit of 300 processes and the sum of the 300 corresponding individual processes must reside in, or derive from, the interactions between the processes. Any non-triviality must arise in, or from, 'the network'. The question therefore is: if we do take those interactions into account, do new properties arise? Is the network special? Or is the behaviour of the entire system not much different from the sum of the behaviour of its macromolecules in isolation?

### Traditional theoretical biology, virtual and e-cells: what may be missing?

There have been quite a few demonstrations of complex properties arising in the interactions of a number of processes. Examples are the oscillations calculated for models of glycolysis. The oscillations did not, and could not, arise in the individual macromolecules; they really required the network [7]. However, this type of calculation did not constitute proof, as they were performed for 'core models'. Such core models contain what might be characteristic properties of the real intracellular networks, but in highly simplified form, and for rather arbitrary parameter values. Hence they show what *might* happen in the intracellular networks, but not what *does* happen. Most theoretical biology shows what might happen, but not what does happen or why it happens.

User-friendly modelling programs and even entire modelling environments, such as the 'virtual cell', have been created for biochemical networks [e.g. 6, 3, 11]. However, contrary to what its name suggests, the 'virtual cell' does not correspond to an image of the cell; it is a valuable collection of software allowing one to model intracellular processes. These collections of software do not suffice to

Copyright © 2003 John Wiley & Sons, Ltd. 

address the properties of the real intracellular networks. The flux balance analysis of the *Escherichia coli* cell generated by Palsson and co-workers [1] serves the important purpose of analysing what happens in the living *E. coli* cell (in terms of flux analysis). However, the modelling methods used in this approach do not contain any other properties of the gene products than that they have the capability to catalyse certain reactions. They do not explain the observed fluxes. Elementary mode analysis [9] *only* uses network stoichiometry and demonstrates which steady-state fluxes might run through the living cell, given some required kinetic properties of the intracellular macromolecules, but does not specify the required properties.

These methods show what does or may happen, but not why it happens. They lack a characteristic of physics that we have not mentioned yet: to explain, not just to analyse. Physics would want to understand, on the basis of the kinetic and thermodynamic properties of the intracellular macromolecules, why these fluxes are as high as they are, and why the phenomena of life occur.

### Attractive silicon models

What makes networks 'live'? From much work in theoretical and mathematical biology we know that self-organization, homeostasis, adaptation, ultrasensitivity, chemotaxis and cell cycling can all arise from biochemical reaction networks, provided that the macromolecules interact in certain ways. Taking an (ideal) metabolic pathway as an example, one may summarize the ways enzymes interact as 'talking' and 'listening'. The 'talking' corresponds to carrying out a chemical conversion at a certain rate. The 'listening' corresponds to enzymes adjusting the rates at which they catalyse their reactions in response to the variations in metabolite concentrations.

The recently initiated Silicon Cell Project [10] differs from the above-mentioned developments in that it has a computer replica in which the macromolecules 'listen' and 'talk' (or whatever else important macromolecules do) *in silico*. It then calculates the behaviour implied by the knowledge we have about the listening and talking. The procedure uses software, not hardware; 'silicon cells' do not correspond to analogue computers but to software replicas of the living cell.

The properties of the macromolecules used in the calculations should be real and based on experimental determinations of those properties themselves. The corresponding experiments typically correspond to *in vitro* enzyme kinetics, or to *in vivo* determinations of the kinetic properties of the individual macromolecules. The kinetic parameters should not be obtained by fitting the parameter values such that the silicon cell behaves just like the real cell. It is in this crucial aspect that silicon cells differ from many existing, 'phenomenological' models. This property of silicon cells is also the one that makes them an important tool to address the issue of whether there is essential new 'physics' in biology.

### Twenty-first century biology: beyond chemistry and physics

Silicon cells should allow one to address this question. Some of the existing silicon cells have suggested that such non-trivial properties indeed arise in realistic networks [e.g. 5, 15]. Although non-trivial, these properties can be down-to-earth, such as the property that the system reaches a metabolic steady state rather than engaging in a metabolic explosion [13]. Whether these properties arise *in silico* depends on the actual magnitude of the kinetic parameters, stressing the importance of silicon cells *vis-à-vis* the already existing 'core models'. What comes from the networks is largely non-trivial. In consequence, biology is not just a complicated special case of physics. Accordingly biology need not, and should not, conform to the paradigm of traditional physics and chemistry. Biology is the science of excellence concerning natural networks and what they bring, and biology may need to grow beyond physics and chemistry.

Silicon cells have only data as input and understanding as output. Hence they classify, as bioinformatics integrates various types of information, we sometimes refer to this approach as 'integrative bioinformatics'. Relative to systems biology, silicon cells have the property that they relate directly to molecular and experimentally determined properties. They may therefore correspond to molecular systems biology.

The fact that silicon cells require precise magnitudes of kinetic parameters as input brings us to a severe limitation to the approach. The processes of

Copyright © 2003 John Wiley & Sons, Ltd.

living cells for which the kinetic properties of all participating macromolecules are known, are rare. Consequently, there are only very few true silicon cells in existence. Indeed most silicon cells do not quite fulfil the above requirements and should be seen as alpha versions of what should constitute the real silicon cell. For their development, silicon cells require close association with precise experimentation, preferably in 'entering the living cell' contexts. We think that the philosophy behind silicon cells and the possibility to create such cells in the wake of functional genomics, will make models of living cells more realistic and dynamic, and therefore attractive and relevant.

### References

1. Covert MW, Schilling CH, Famili I, *et al.* 2001. Metabolic modeling of microbial strains in silico. *Trends Biochem Sci* **26**: 179–186.
2. Gasch AP, Spellman PT, Kao CM, *et al.* 2000. Genomic expression programs in the response of yeast cells to environmental changes. *Mol Biol Cell* **11**: 4241–4257.
3. Gepasi: **http://www.gepasi.org/**.
4. Hutchison CA, Peterson SN, Gill SR, *et al.* 1999. Global transposon mutagenesis and a minimal *Mycoplasma* genome. *Science* **286**: 2165–2169.
5. Kholodenko BN, Demin OV, Moehren G, Hoek JB. 1999. Quantification of short term signaling by the epidermal growth factor receptor. *J Biol Chem* **274**: 30 169–30 181.
6. Mendes P. 1997. Biochemistry by numbers: simulation of biochemical pathways with Gepasi 3. *Trends Biochem Sci* **22**: 361–363.
7. Prigogine I, Nicolis G. 1971. Biological order, structure and instabilities. *Q Rev Biophys* **4**: 107–148.
8. Rosen R. 1991. *Life Itself.* Columbia University Press: New York.
9. Schuster S, Fell DA, Dandekar T. 2000. A general definition of metabolic pathways useful for systematic organization and analysis of complex metabolic networks. *Nature Biotechnol* **18**: 326–332.
10. Silicon Cell Project: **http://www.siliconcell.net**.
11. Slepchenko BM, Schaff JC, Carson JH, Loew LM. 2002. Computational cell biology: spatiotemporal simulation of cellular events. *Ann Rev Biophys Biomol Struct.* **31**: 423–441.
12. Spellman PT, Sherlock G, Zhang MQ, *et al.* 1998. Comprehensive identification of cell cycle-regulated genes of the yeast *Saccharomyces cerevisiae* by microarray hybridization. *Mol Biol Cell* **9**: 3273–3297.
13. Teusink B, Walsh MC, van Dam K, Westerhoff HV. 1998. The danger of metabolic pathways with turbo design. *Trends Biochem Sci* **23**: 162–169.
14. Wallace DC, Morowitz HJ. 1973. Genome size and evolution. *Chromosoma* **40**: 121–126.
15. Westerhoff HV, Getz WM, van Verseveld HW, Hofmeyr JHS, Snoep JL. 2002. Bioinformatics, cellular flows, and calculation. *Ernst Scher Found Symp* **38**: 221–243.

Copyright © 2003 John Wiley & Sons, Ltd.
